# Plants in alcoholic beverages on the Croatian islands, with special reference to rakija travarica

**DOI:** 10.1186/s13002-019-0332-1

**Published:** 2019-11-05

**Authors:** Łukasz Łuczaj, Marija Jug-Dujaković, Katija Dolina, Ivana Vitasović-Kosić

**Affiliations:** 1 Institute of Biology and Biotechnology, University of Rzeszów, ul. Pigonia 1, 35-310 Rzeszów, Poland; 2 Institute for Adriatic Crops and Karst Reclamation, Put Duilova 11, 21000 Split, Croatia; 3 Institute for Marine and Coastal Research, University of Dubrovnik, Kneza Damjana Jude 12, PO Box 83, 20000 Dubrovnik, Croatia; 4 Department of Agricultural Botany, Faculty of Agriculture, University of Zagreb, Svetošimunska cesta 25, 10000 Zagreb, Croatia

**Keywords:** Travarica, Ethnobotany, Culinary independence, Alcohol, Grappa, Mediterranean cuisine

## Abstract

**Background:**

This paper aims to record the species used for flavouring and making alcoholic drinks, mainly rakija, on the islands of the Adriatic (Croatia).

**Methods:**

Our data comes from 295 interviews performed on 36 islands, in both the Dalmatian and Kvarner areas of the Adriatic.

**Results:**

Altogether, 114 species are used—46% from wild locations only, 15% both wild and cultivated, 38% only cultivated and two species are imported.

The most common local alcohol is wine, made without spices, but grape pomace distillate is often flavoured with single or mixed species. The mix is called *travarica.* The most commonly used species are *Foeniculum vulgare* Mill., *Myrtus communis* L., *Salvia officinalis* L., *Ruta graveolens* L., *Juniperus oxycedrus* L., *Ceratonia siliqua* L., *Juglans regia* L., *Citrus* spp., *Ficus carica* L., *Laurus nobilis* L., *Rosmarinus officinalis* L., *Artemisia absinthium* L., *Rosa centifolia* L., *Mentha* × *piperita* L. and *M. spicata* L. Unfortunately, the widespread phenomenon of distilling *Arbutus unedo* L. fruits and fermenting *Juniperus* ‘wine’ is now extinct. Apart from grapes, the only commonly distilled fruit now is *Ficus carica.*

**Conclusions:**

It is striking that nearly all the plants are either wild or cultivated locally, which, in addition to the fact that the alcohol is made locally, shows the incredible local culinary self-sufficiency of the area. The number of species used is also very impressive.

## Background

Regional alcoholic drinks are an important part of local culinary identity, not only in Europe [1–15]. In some parts of Europe, this identity is associated with local wines; in others, it is beer or high-percentage alcohol. Plants used for flavouring alcoholic drinks in some parts of Europe have been hardly documented, a rare example being Tuscany, Italy [1, 2].

It is worth mentioning that alcoholic drinks should not only be seen as intoxicating beverages or gourmet products, but also as components of local food security strategies, as pointed out by Madej et al. [9]. Excess fruit, when turned into an alcoholic drink, can be stored for a long period of time in its new form. Additionally, the drink preserves some of the caloric value or other nutritional qualities of the original product from which it derives. Moreover, flavoured alcoholic drinks are strongly embedded in the tradition of herbalism. Alcohol is a very good material for the chemical extraction of medicinally active chemicals; hence, its use for making tinctures has been widespread since antiquity [14–17].

The coast of Croatia has recently been the subject of intense ethnobotanical investigation [18–27], mainly by the authors of this paper [18–25].

Most of the studies were focused on wild foods, though medicinal plant use in Istria and inland Dalmatia was also recorded. Wine is the dominant locally produced alcoholic drink in Croatia, hence a large variety of grape cultivars and types of wine are known. After wine-making, the remaining pressed grape stalks and pulp (i.e. pomace) are distilled into a grappa-type spirit called *rakija.*

Drinking rakija is also a social phenomenon. It is nearly always offered to guests and is drunk on many important occasions such as weddings and funerals. It is also consumed as a simple digestive. Rakija is made in nearly every rural household in coastal Croatia. However, since joining the European Union in 2013, there have been constraints on the selling of home-made spirits, and a special licence is now required.

Rakija is actually a term meaning all spirits which originate from the fermentation of fruits. In this paper, we will use the term rakija and grappa interchangeably. The word rakija comes from the Turkish *raki* (originally a word of Middle Eastern origin) and is commonly used not only in Croatia, but in other Balkan countries, which used to be a part of the Ottoman Empire [28–31]. On the other hand, it is argued that the making of rakija in the neighbouring Bosnia may have been brought from Dalmatia where spirits were made earlier than in the western part of the Ottoman Empire [31], the Venetians being pioneers of the production of aromatic spirits in Europe [15]. In Croatia, strong spirits are often flavoured with herbs and fruits. In spite of the fact that this tradition is widespread, as yet no monograph documents this phenomenon in its entirety. The only sources on flavoured rakija are a few popular handbooks documenting how to make it [32, 33]. These however tend to contain species promoted by the author rather than documenting local practices or traditions. In order to fill this gap, we aimed to record the traditions of flavouring alcohol on the islands of the Croatian coast. Croatian islands are a good model for studying island biogeography principles [34] in ethnobotany. The theory of island biogeography [35] has hardly been applied at all in ethnobotany, our previous study on the use of wild vegetables on the Croatian islands constituting the first systematic study of this kind [34]. We found hardly any correlations between island geographical characteristics (such as size, population, flora and isolation from the mainland) and the number of wild vegetables used, apart from a generally increasing richness from the northwest to the southeast. However, in the previous paper, we only looked at the 15 largest islands, whereas in this study, we have data from nearly all the inhabited islands, including the most sparsely inhabited, presenting a greater variety of island sizes.

The main aim of our study was to compile a list of species used to flavour alcohol and determine whether there are any major differences within the study area. The islands of Croatia form one of the largest island archipelagos in Europe. We expected that the high diversity of dialects, relative isolation of local populations and abundance of aromatic Mediterranean plants would result in a high number of taxa used.

## Methods

In the Croatian part of the Adriatic there are 1151 islands, islets and reefs, and 80 additional reefs which periodically appear above sea level, depending on the tides. Only 47 islands are inhabited, in the sense that at least one person resides there. Sixty Croatian islands have coastlines longer than 10 km, while 653 islets have coastlines shorter than 10 km, but with developed soil and vegetation. The highest elevation occurs on Brač (778 m a.s.l.). The climate of the area is mainly Mediterranean. According to the Köppen classification system, the coast and islands of the eastern Adriatic side belong to three Mediterranean climate types: (1) Cfsax, (2) Csax and (3) Csa. According to Mazzoleni et al., the entire area belongs to the IV3 climate type [36]. The potential vegetation over the largest areas of the studied islands is made up of forests dominated by *Quercus ilex* (belonging to the phytosociological class of *Quercetea ilicis*). On the Kvarner islands and the inland hills of larger islands, the potential natural vegetation is made up of downy oak forests (*Quercetalia pubescentis*) [37, 38].

The traditional food systems of the islands were based on utilising marine resources, mainly fish, and the cultivation of olives and grapes supplemented by grains, legumes and brassicas, along with animal husbandry, particularly in off-coast locations. Nowadays, the tourist industry is the dominant source of income.

**Fig. 1:**
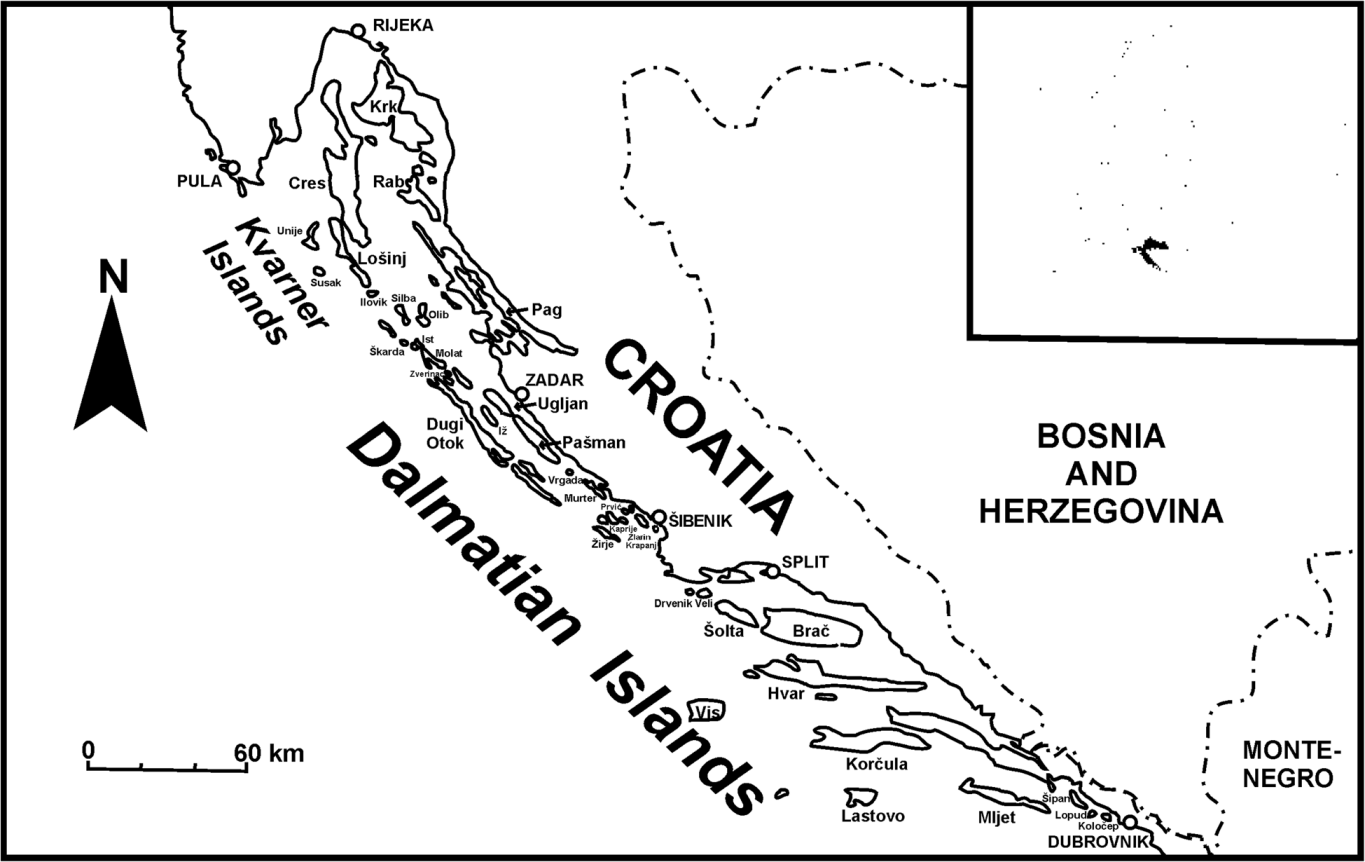
Map of the Adriatic Sea and southern Croatia showing the studied islands (lower case font) and major cities (in capitals)

This study forms part of a larger study on the ethnobotany of the Adriatic islands, from which only data about wild vegetable use has so far been published [34]. The research was performed between 2013 and 2018, with most interviews carried out in 2016 and 2017. One of the study questions enquired as to which plants were used to make flavoured rakija, liqueurs and other alcoholic drinks. The data in the spreadsheet come from 295 interviews (279 unpublished and 16 interviews from which data had already been published in an earlier paper about the island of Krk [20]). The mean age of respondents was 69 (median 70, minimum 30, maximum 96; 62% female, 38% male). We managed to interview people from 36 out of the 47 officially inhabited islands, including the 15 largest islands, with surface areas of over 40 km^2^ (Fig. 1, Table 1).

**Table 1:** Islands where the interviews were conducted

	Number of interviews	Area (km^2^)	Population	Flora	Longitude (degrees E)	Isolation (minimum km distance from mainland)
Kvarner						
Krk	16	405	19,383	1170	14.61	0.8
Cres	14	406	3079	1250	14.39	5
Rab	14	86	9328	800	14.77	2
Lošinj	13	74	7587	1300	14.43	29
Ilovik	5	5	104	n.d.	14.55	56
Unije	5	17	90	629	14.25	28
Susak	3	4	188	400	14.30	41
Zadar Archipelago (Northern Dalmatia)						
Pag	15	284	9059	650	15.04	0.4
Pašman	13	60	2845	629	15.34	2
Ugljan	10	51	6049	n.d.	15.17	4
Dugi Otok	9	113	1655	540	15.03	16
Molat	9	22	207	308	14.83	17
Vrgada	5	2	242	462	15.50	3
Iž	3	16	557	n.d.	15.11	12
Olib	3	26	147	465	14.79	25
Silba	3	14	265	516	14.69	32
Ist	2	10	202	438	14.76	20
Skarda	1	4	0	237	14.71	32
Zverinac	1	4	48	n.d.	14.92	18
Šibenik Archipelago (Central Dalmatia)						
Murter	6	18	5060	664	15.62	0.1
Žirje	6	15	124	542	15.67	12
Kaprije	5	7	143	278	15.71	5
Krapanj	5	0.4	237	259	15.91	0.3
Prvić	5	2	453	267	15.80	0.8
Zlarin	5	8	276	444	15.85	2
Split Archipelago (Central Dalmatia)						
Hvar	16	297	11,077	1046	16.8	4
Šolta	16	58	1700	267	16.31	15
Brač	12	395	13,956	750	16.66	5
Vis	11	90	3445	598	16.16	43
Drvenik Veli	5			n.d.	16.15	2
Dubrovnik Archipelago (Southern Dalmatia)						
Korčula	22	271	15,522	858	16.93	1
Mljet	14	98	1088	712	17.55	8
Lastovo	10	41	792	678	16.87	26
Šipan	5	16	436	570	17.88	2
Koločep (Kalamota)	4	2	294	446	18.00	1
Lopud	4	4	269	429	17.95	2
TOTAL	295					

*n.d.* no data available

We applied the standard methods of ethnobotany: in-depth semi-structured interviews starting from freelisting and supplemented if possible by walks around the places where the respondents gathered plants and could identify the supplied names. Two of the authors of this paper were brought up on the coast of Dalmatia (M.J.D. – Split; K.D. – Dubrovnik) and had frequently visited many of the studied islands since childhood. Some key informants were also selected by walks through the fields with people who claimed that they still collected wild food plants or made well-flavoured rakija. The interviews were performed in Croatian, the native language of the inhabitants. The interviews concerned different aspects of plant use, but here, we present data only on alcoholic beverages, and only with respondents who possessed such knowledge. We asked the general question, which plants (wild or cultivated) are used to flavour alcoholic drinks? We made efforts to cover the whole island evenly and recruit informants from as many different villages as possible. The informants were chosen from people who were born on the islands and had their ancestry there.

The number of species in the island floras was extracted from data gathered by Nikolić et al. [36], supplemented by the flora of Pašman [39], Vrgada [40], Olib [41], and Ist and Skarda [42]. The isolation of the island was measured as the distance (km) between the mainland and the part of the island closest to it. The population data were taken from the Statistical Yearbook of 2015 [43].

Plants were identified using standard floras available in this area of Europe, including Nikolić’s guide for the identification of Croatian flora [44], Pignatti’s Flora of Italy [45] and the Flora Croatica Database [46]. Plant names were updated to be consistent with The Plant List [47]. Voucher specimens were collected on the islands where they were used, usually with the assistance of the respondents. For the place of deposition, see the “Availability of data and materials” section.

In some of our analyses, we divided the islands into five groups corresponding to their administrative location in five regions (‘županija’): (1) Kvarner islands, (2) Zadar islands (North Dalmatian Islands), (3) Šibenik islands and (4) Split islands (Central Dalmatian Islands), and (5) Dubrovnik islands (South Dalmatian Islands). We grouped nearby islands, as there is usually cultural exchange between neighbouring islands, and the number of interviews from each island was very uneven.

Statistical analysis was performed using open-access PAST software [48]. The normality of the distribution of variables was tested with the Shapiro-Wilk test. All the variables had a normal distribution. A matrix of Pearson correlation coefficients for all pairs of variables was created (the variables used were number of species listed by an informant, gender of informant (male = 1, female 0), age, area of the island, population, isolation from mainland, number of species in the vascular flora and longitude).

To visualise the similarity of species lists in the studied regions and in larger islands, we used a numeral taxonomy dendrogram obtained by clustering. We used the most common method of clustering, i.e. Unweighted Pair Group Method with Arithmetic Mean (UPGMA), using Euclidean distance [49, 50].

## Results and discussion

**Fig. 2:**
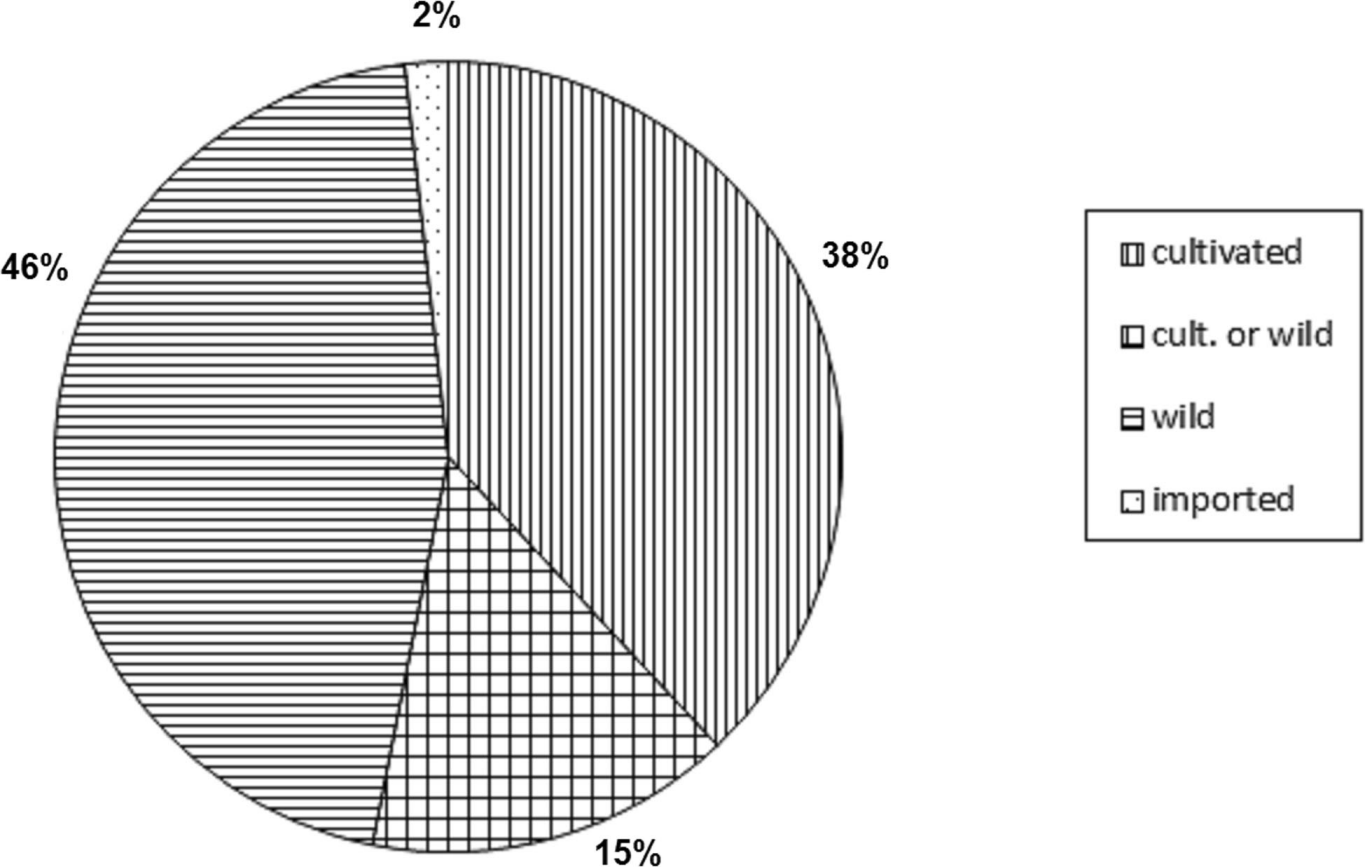
Proportions of wild, cultivated and imported species used

**Fig. 3:**
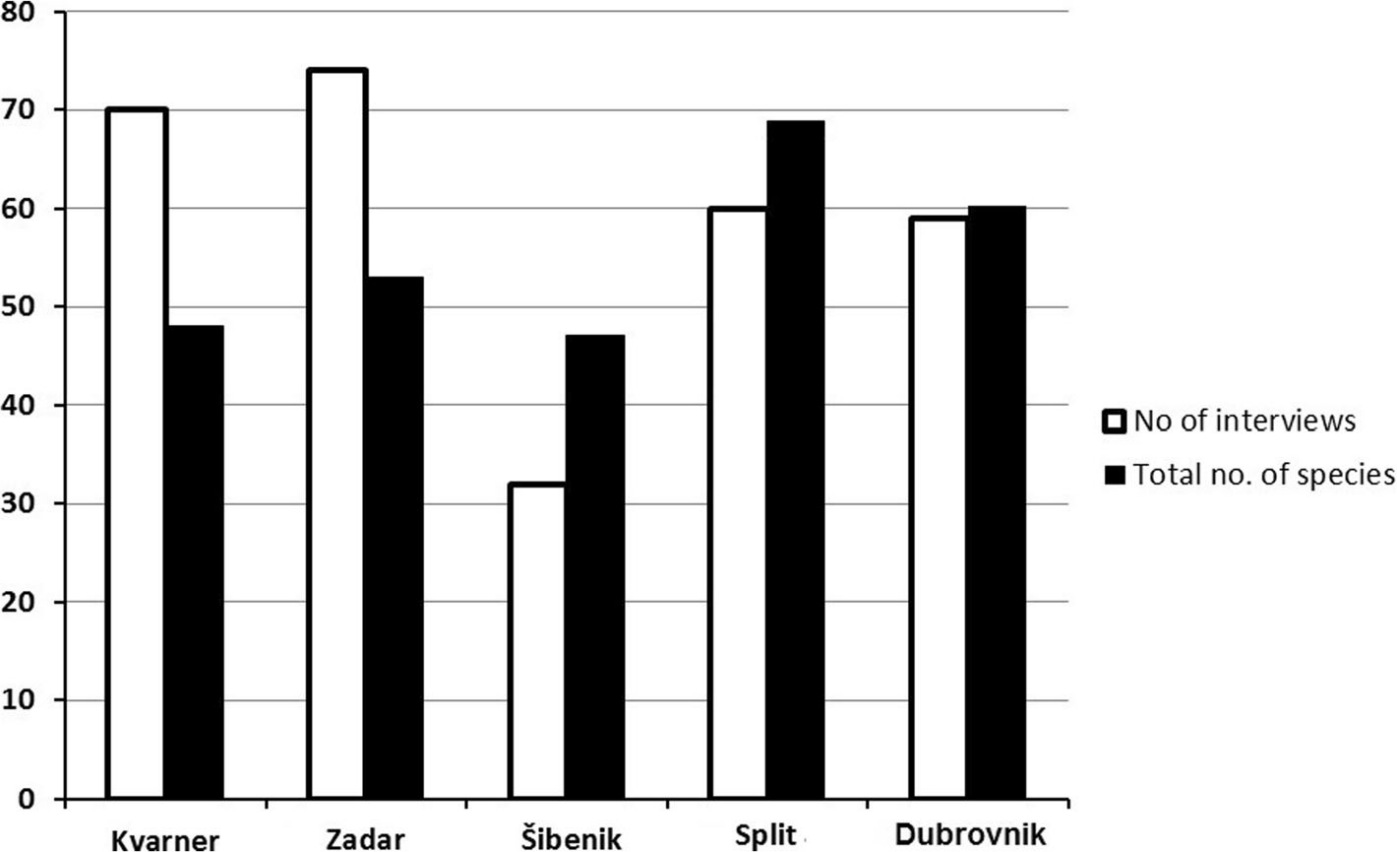
Total number of species recorded in different parts of the Adriatic

**Fig. 4:**
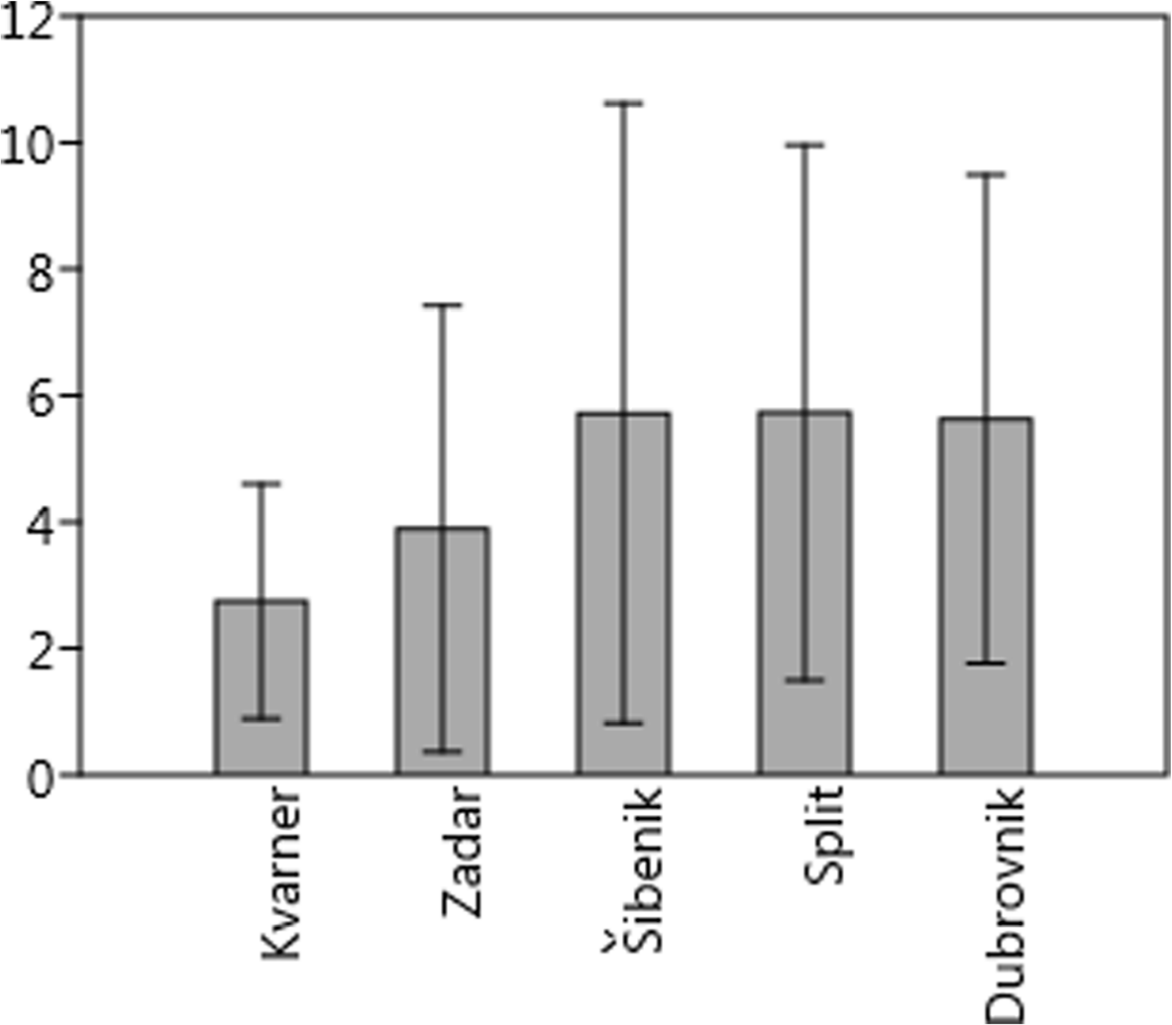
Mean number (and standard deviation) of species mentioned per interview

Altogether, 114 species of plants from 38 botanical families are involved in the production or flavouring of alcoholic drinks (Table 2, Figs. 2, 3, and 4). The largest families represented were Lamiaceae (19 species), Rosaceae (18), Asteraceae (12) and Rutaceae (6). The largest category is composed of wild plants (52 species, 46% of species). If we add the species which are gathered both from wild localities and from gardens (17 species), they constitute over half of the species list (Fig. 2). Forty-three species are exclusively cultivated, and only two species from one genus (coffee) are imported. The most important plant is of course grape (*Vitis*), which was not usually mentioned in interviews, regarded as being too obvious since wine is a basic everyday drink in southern Croatia. The remnants from the pressing of wine must, called ‘pomace’ in English (and *drop* in Croatian), are distilled into rakija. Rakija is a general term for any liquors distilled from fruits or their juices. Specifically, *lozovača* is the one made from grapes. Spirits have also been distilled from dried figs *Ficus carica* L. (the drink is called *smokovača*) and strawberry tree *Arbutus unedo* L. fruits (the latter species ceased to be used in 1950–1960s) or more rarely plum species *šljivovica* (*Prunus* spp.). The distillation of dried figs is still common on a very small scale (and the product is highly prized by alcohol gourmands). On the other hand, we have not found a single contemporary producer of strawberry tree distillate. At one time, this was so common that people from the Šibenik islands brought boats full of strawberry tree fruits and sold them in the Šibenik market.

**Table 2:** The list of species used to make alcoholic drinks in the Adriatic Islands

	Kvarner	Zadar Arch.	Šibenik Arch.	Split Arch.	Dubrovnik Arch.	Total		Distribution	Local names*	1	2	3	Medicinal uses
No. of interviews	70	74	32	60	59	295							
Total no. of species	48	53	47	68	60	114							
No. of species per interview	2.7	3.9	5.7	5.8	5.5	4.5							
*Achillea millefolium* L. (Asteraceae) ZAGR39685				1	1	2	cw	Korčula, Šolta	stolisnik	m	r	fl	
*Aesculus hippocastanum* L. (Sapindaceae)	1	1				2	cw	Lošinj	divlji marun	s	r	fr	Only against rheumatism
*Agrimonia eupatoria* L. (Rosaceae) WA66922		1				1	w	Dugi Otok	divlja melisa	m	r	ae	
*Ajuga chamaepitys* (L.) Schreb. (Lamiaceae) WA66388		1				1	w	Ugljan	trava iva	m	r	ae	
*Aloe* sp. (Asphodelaceae)					1	1	cw	Lastovo	Aloe vera	s	r	l	
*Aloysia citriodora* Palau (Verbenaceae) WA66484			1		4	5	cw	mainly Korčula, also Zlarin	alviz, alviza, alviža, also: bela luiđa ZL	m	r	ae	Pregnant women should avoid it
*Anethum graveolens* L. (Apiaceae) WA66391		4			1	5	c	Pag, Mljet, Vrgada	anita PG, kopar ML, anis VR	m	r	ae, l	
*Arbutus unedo* L. (Ericaceae) WA66321	7	22	11	26	21	87	w	throughout, more in the south	fruit: maginja, magunja, manjiga, meginja, mogunja, also: plančići UN; plant: planika	s/m	rb, r	fr	Rakija used to be commonly distilled from it until 1960s, now only addition to mixed rakijas
*Artemisia abrotanum* L. (Asteraceae)					3	3	c	Lastovo, Korčula	broda KO, srčano zelje LA	m	r	ae, l	Only medicinal to induce menstruation
*Artemisia absinthium* L. (Asteraceae) WA66375	6	12	7	11	3	39	cw	throughout	pelin, also: asinac, ašinac, osjenač KO, domaći pelin, vrtlarski pelin ŠO	m/s	r	ae, l	For digestion, stomach ache, against worms
*Artemisia caerulescens* L. (Asteraceae) WA66473	2					2	w	Cres	morski pelin, divlji pelin, pelino	s	r	ae, l	Against worms
*Arum italicum* Mill. (Araceae) WA66915				1		1	w	Brač	žuminac	m	r	?	
*Bellis perennis* L. (Asteraceae)	1					1	w	Rab	divlja tratinčica	s/m	r	ae	Medicinal rakija for massaging swollen legs
*Calendula officinalis* L. (Asteraceae) WA66413	1					1	c	Rab	neven	s/m	r	fl	Medicinal rakija for massaging swollen legs
*Carduus* cf. *nutans* L. (Asteraceae)		1				1	w	Molat	sikavica	m	r	fl	Also against stomach ache
*Celtis australis* L. (Cannabaceae) WA66311	1	4	3	5	5	18	c	Throughout	fafarinka, fararikula, kostanja, kostela, košćela, košćila, kopriva, koprivić, koprva	s	r	fr	
*Centaurium erythraea* Rafn (Gentianaceae) WA66924	1	2		1		4	w	Throughout	kičica	m	r	ae	Stimulating appetite
*Ceratonia siliqua* L. (Fabaceae) WA66455	1	11	6	11	25	54	c	More in the south	karuba, korub, rogač, also: VI jubica	m/s	r	fr	Good for stomach, against diarrhoea
*Cistus* × *incanus* L. (Cistaceae) WA66353				4		4	w	Vis	divji pelin, carveni pelin	m	r	l	
*Cistus monspeliensis* L. (Cistaceae) WA66403				1		1		Vis	slipavi pelin	m	r	l	
*Cistus salviifolius* L. (Cistaceae) WA66449				1		1		Vis	pelin, bili pelin	m	r	l	
*Citrus* × *aurantium* L. (Rutaceae) WA71108					10	10	c	Only south	divlja naranča, gorka naranča, limon, ljuta naranča, naranča	m	r	p, l, fr	
*Citrus japonica* Thunb. (Rutaceae)				1		1	c	Drvenik Veli	kumkvat	s/m	r	fr	
*Citrus limon* (L.) Osbeck (Rutaceae) WA66464	5	7	2	16	13	43	c	Throughout more in the south	limon, limun	s/m	r	p, l, fr	Fruit used to make limončelo or fruit peel/leaves added to mixed travarica
*Citrus reticulata* Blanco (Rutaceae) WA66333		2	1	2	3	8	c	Throughout more in the south	mandarina, mandarinka, (slatka) naranča	s/m	r	p, l	Fruit used to make a single-species sweet liqueur or fruit peel/leaves added to mixed travarica
*Citrus sinensis* (L.) Osbeck (Rutaceae)		1	1	13		15	c	Throughout	naranča	s/m	r	p, l, fr	Fruit used to make a single-species sweet liqueur or fruit peel/leaves added to mixed travarica
*Clinopodium nepeta* (L.) Kuntze (Lamiaceae) WA66943	1	5	1	4	4	15	w	Throughout	divlja menta, divlja metvica, menta, metvica, also: ošji mravinac KO, šošenica VI	m	r	ae	
*Coffea arabica* L. (Rubiaceae) and *C. liberica* Hiern		1	2	1		4	i	Throughout	mainly: kava, also: kafa	m	r	s	
*Cornus mas* L. (Cornaceae) WA66480	1					1	w	Cres	drenjula	s	r	fr	
*Crataegus azarolus* L. (Rosaceae) WA66306					1	1	c	Korčula	glog	s/m	r	fr	
*Crataegus monogyna* Jacq. (Rosaceae) WA66394	1	1				2	w	Krk, Pag	glog crveni, also: jabučica PA	s/m	r	fr	
*Crocus biflorus* ssp. *weldenii* (Hoppe & Fürnr.) K.Richt. (Iridaceae)				1		1	w	Šolta	šafran	m	r	st	
*Crocus sativus* L. (Iridaceae)					5	5	c	Korčula, Koločep	šafran		r	st	
*Cydonia oblonga* Mill. (Rosaceae)				1		1	c	Vis	dunja	m	r	fr	
*Dioscorea communis* (L.) Caddick & Wilkin (Dioscoreaceae) ZAGR39307	1					1	w	Krk	blušt	s	r	ae	Only medicinal, to treat swollen legs
*Dittrichia viscosa* (L.) Greuter (Asteraceae) WA66302		2		2		4	w	Dugi Otok, Vis	lipavi pelin, pelin, tavari bušinjak, tovareći bušinak	m	r	ae	
*Elymus repens* (L.) Gould (Poaceae) WA71140			1			1	w	Prvić	pirika	m	r	u	
*Erica arborea* L. (Ericaceae) WA66432		1		2		3	w	Dugi Otok, Šolta, Vis	vris, vrisak	m	r	ae	
*Eriobotrya japonica* (Thunb.) Lindl. (Rosaceae)	3	1			8	12	c	throughout	nespola, nešpola, nešpula	s	r	fr	
*Eucalyptus globulus* Labill. (Myrtaceae)	1					1	c	Lošinj	eukaliptus	m	r	l	
*Ficus carica* L. (Moraceae)	7	10	9	6	8	39	c	Throughout	divlja smokva, smokva, also: smokvenjak, smokvenci LO, smukva UG	s/m	rb, r	fr	
*Foeniculum vulgare* Mill. (Apiaceae) WA66401	17	24	15	31	19	106	w	Throughout	divji koromač, komorač, koromac, koromač, kromač, morač, koromacić	m/s	r	ae	
*Geranium macrorrhizum* L. (Geraniaceae)					1	1	c	Korčula	kanela	m	r	l	
*Helichrysum italicum* (Roth) G.Don (Asteraceae) WA66460		6	2	2	2	12	w	Throughout	cmilj, magriž, smilj, smilje, also: kadila PA	m	r	ae	
*Hypericum perforatum* L. (Hypericaceae) WA66461		7	4	7	3	21	w	Throughout	gospino cvićje, gospina trava, kantarion	m	r	ae, fl	Stomachic
*Juglans regia* L. (Juglandaceae) ZAGR39700	4	12	14	10	5	45	c	Throughout	orah, orih	s	r	im	Stomachic
*Juniperus macrocarpa* Sm. (Cupressaceae) WA66907	10	2	8	4		24	w	Throughout	bujač, kleka, ljuskovac, loskač, luskač, luskavac, luskavica, luškač, luškavica, pucalinka, pukomnjari, smrič, smriče, smričkalj, smreka, smrijek, smrika (fr smričići), also: slukovci RA, šišarke [name of fruits] ZL	m	r, w	fr	Used widely, but in Cres and Rab made into a kind of wine
*Juniperus oxycedrus* L. (Cupressaceae) WA66332	17	9	7	17	9	59	w	Throughout	badljač, badovi smric, badovi smrik, bujač, badžic, (divlja) smreka, luskač, luskovica, luškač, pukinja, smirčić, smrča, smrekujići, smrič, smrička, smrijek, smrika, smrjek, smrjeka, smriška, šmrikuići, šmirka, šmrika, šmrkujić/ki, šišarke [name of fruits], also: pomele CR	m	r, w	fr	Used widely, but in Cres and Rab made into a kind of wine
*Juniperus phoenicea* L. (Cupressaceae) WA66326	1	1		2		4	w	Ilovik, Hvar, Dugi Otok	brika, gluhač, luskavac, smrič	m	r	fr	
*Laurus nobilis* L. (Lauraceae) WA66310	9	8	5	12	4	38	cw	Throughout	javor, javorika (fruit: javorčić), lovor, lovorika, also: laurano CR	m/s	r	l, fr	Although usually leaves are added to mixed travarica a sweet liqueur made from fruits is made in Cres and Lošinj
*Lavandula* × *intermedia* Emeric ex Loisel. (Lamiaceae) WA71147 and *L. angustifolia* Mill. WA66937		2	1	2	3	8	c	Throughout more in the south	lavanda, levanda	m	r	ae	
*Malus domestica* Borkh. (Rosaceae) WA66945	1	2	1		1	5	c	Throughout	divlja jabuka, jabuka	m	r	fr	
*Matricaria chamomilla* L. (Asteraceae) WA66467		2	1		1	4	cw	Molat, Zlarin, Korčula	kamilica, kamomila	m	r	fl	
*Matthiola incana* (L.) R.Br. (Brassicaceae) WA66930			1			1	c	Žirje	domaća viola	m	r	fl	
*Melissa officinalis* L. (Lamiaceae) WA66495			2	3	1	6	c	Throughout	čejena ljubica, čelinja lubica, ljubica, matičnjak, melisa, pčelinja ljubica	m	r	ae	
*Mentha* × *piperita* L. (Lamiaceae) WA66402 and *M*. *spicata* L. WA66348	2	4	4	4	11	25	c	Throughout more in the south	divlja metica, menta, menta alkoholna, menta divlja, metvica, minta, murtela, also: guin träva, kamilica pitoma MU, sarsa ŠO	m	r	ae	
*Micromeria graeca* (L.) Benth. ex Rchb. (Lamiaceae)				1		1	w	Vis	mažuron	m	r	ae	
*Micromeria juliana* (L.) Benth. ex Rchb. (Lamiaceae) WA66396				2		2	w	Brač	bresina, čubar	m	r	ae	
*Morus nigra* L. (Moraceae) and *M. alba* L. ZAGR39WA664		1		1	2	5	c	Throughout	dud, murva	s	r	fr	
*Myrtus communis* L. (Myrtaceae) WA66307	15	29	7	22	28	101	w	Throughout	marka (fruit) martovica (plant), marta, martići (fr), martina, mirta, markići (fr) mirta (plant), mrča, mrčakinja, mrčica, mrčka, mrka, mrta, mrtakinja, mrtica, mrtina, mrtovnica, murta, murtići (fr), smrške, also: jurovika KO, zoba LA	s	r	fr	
*Ocimum basilicum* L. (Lamiaceae) WA71155			2	2	3	7	c	Throughout	bosiljak, murtela	m	r	ae	
*Olea europaea* L. (Oleaceae)	3	4	1	2	1	11	cw	A new fashion	maslina	s/m	r	fr	
*Origanum majorana* L. (Lamiaceae) WA66443			3	1	11	15	c	Korčula, Mljet, Šolta, Zlarin	mažuran, mažurana, also: metvica KO	m	r	ae	
*Origanum vulgare ssp. viridulum* (Martrin-Donos) Nyman (Lamiaceae) WA71146	1		1	2	3	7	w	Throughout	origano, oregano, mravinac	m	r	ae	
*Paliurus spina-christi* Mill. (Rhamnaceae) WA66316	1		2		1	4	w	Throughout	drač, drača	m	r	im	
*Papaver rhoeas* L. (Papaveraceae) WA66381	1					1	w	Krk	mak	m	r	fl	
*Parietaria judaica* L. (Urticaceae) WA66338	2	1				3	w	Cres, Pag, Rab	lepek CR, šćir PG, šćirenica RB	s	r	ae	Only medicinal for kidneys, bladder, prostate, veins
*Pelargonium odoratissimum* (L.) L’Hér. (Geraniaceae) WA71132					1	1	c	Korčula	barbaroza	m	r	l	
*Phillyrea latifolia* L. (Oleaceae) WA66446				1		1	w	Vis	zelenika	m	r	fr	
*Pimpinella anisum* L. (Apiaceae)	1				2	3	c	Cres, Korčula, Mljet	anis, aniš, aniž	s/m	r	fr	
*Pinus halepensis* Mill. (Pinaceae) WA71148					1	1	w	Korčula	bor	s	r	l	
*Pinus pinea* L. (Pinaceae) WA66399				1		1	c	Vis	pinj	s	r	l	
*Pistacia lentiscus* L. (Anacardiaceae) WA66383	1			3		4	w	Brač, Šolta, Unije, Vis	lonjstik UN, smarška VS, smrča ŠO, smrčica BR	m	r	fr	
*Plantago lanceolata* L. (Plantaginaceae) WA71162				1		1	w	Korčula	trputac	m	r	l	
*Prunus avium* (L.) L. (Rosaceae) WA66469	1	1		1		3	c	Cres, Ugljan, Vis	divja višnja VS, trešnja CR, trišnja UG	s	r	fr	
*Prunus cerasus* L. (Rosaceae)		8	6	5	5	24	c	Throughout	maraska, maraška, višnja	s	r	fr	
*Prunus domestica* L. (Rosaceae) ZAGR39690 and *P. cerasifera* Ehrh.				1	2	3	cw	Korčula, Šolta	sliva	s	rb, r	fr	
*Prunus dulcis* (Mill.) D.A.Webb (Rosaceae)		3	2	2	3	10	c	Throughout	badem, bajam, mindel, mindela, mjendul	m	r	pe	
*Prunus mahaleb* L. (Rosaceae) WA66430				1		1	w	Šolta	rašeljka	s	r	fr	
*Prunus persica* (L.) Batsch (Rosaceae) WA66421	1		3		1	5	cw	Cres, Lastovo, Zlarin, Žirje	breskva, glodžanica ŽI, glodalica, praska, voćka ZL, praskva CR	s	r	fr	
*Prunus spinosa* L. (Rosaceae) WA66367	3	6	2	1	1	13	w	Throughout	divlji trn, tarno, torno, trn, trnina, trnjikula, trnjovača, also: brambula OL	s	r	fr	
*Punica granatum* L. (Lythraceae) WA66478	1		3		1	5	cw	Rab, Krapanj, Zlarin	mogranj RB, nar KR, šipak ZL	m	r	pe	
*Pyrus amygdaliformis* Vill. (Rosaceae) WA66904		2				2	w	Pag	divlja kruškica, krušvica	s	r	fr	
*Pyrus communis* L. (Rosaceae)					2	2	c	Korčula, Šipan	kruška	s	r	fr	
*Rhamnus alaternus* L. (Rhamnaceae) WA66920				3		3	w	Vis	kokočika	m	r	fr	
*Rosa canina* L. (Rosaceae) WA66309 and *R. sempervirens* L. WA66323	4	3	1	1	1	10	w	Throughout	divlja ruža, šipak, šipurak, šepurika	m	r	fr	
*Rosa centifolia* L. (Rosaceae) WA66941		1	10	5	14	30	c	Commonly but most popular on Korčula	ruža, also: roza iz kući KO	s	r	fr	
*Rosmarinus officinalis* L. (Lamiaceae) WA66366	3	9	4	12	9	37	cw	Throughout more in the south	ružmarin, lucmarin, also: rusmarin, ruzmarin, zimorod, zumorod, zumrod, zemorod HV	m	r	ae	
*Rubus ulmifolius* Schott (Rosaceae) ZAGR39711	1	2	2		10	15	w	Throughout	kupina, jagoda, also: kupjena ŠI, kupjenica LA, zrača KO	s	r, w	fr	
*Ruscus aculeatus* L. (Asparagaceae) WA66369	1					1	w	Lošinj	leprinac	s	r	fr	Liqueur from fruits
*Ruta graveolens* L. (Rutaceae) WA66380	28	16	7	17	5	73	cw	Throughout	ruta, also: rutnjak IŽ, rutva KR, LA, rutvica ŽI, trava ruta VI, PA	s/m	r	ae	To cure infertility, lack of appetite, protect from evil eye, against worms
*Salvia officinalis* L. (Lamiaceae) WA66459	6	13	10	28	17	74	cw	Throughout	kadulja, kaduja, slavulja, slavuja, also: kuš CR, KR, LO, pelin LO, ML, ŠI	m	r	l, fl	
*Salvia verbenaca* L. (Lamiaceae)				1		1	w	Vis	[no name recorded]	m	r	ae	
*Sambucus nigra* L. (Adoxaceae) WA66415				2	1	3	c	Throughout	bazga, also: sambuk KO	s	r	fl	
*Santolina chamaecyparissus* L. (Asteraceae) WA71111				1		1	c	Vis	trovo od crvih	s	r	ae	Only medicinal against worms
*Satureja montana* L. (Lamiaceae) WA66397		8	1	7	3	19	w	Throughout	vrisak, also: kidež ML, meta DU, vris BR	m	r	ae	
*Satureja visianii* Šilić (Lamiaceae) WA66385 (probably usually not distinguished from S. montana)							w	Korčula	vrisak	m	r	ae	
*Sedum* cf. *telephium* L. s.l. (Crassulaceae) WA66494				1		1	c	Vis	žednjak	m	r	l	
*Sorbus domestica* L. (Rosaceae) WA66319			2	4	3	9	cw	Throughout	oskoruša, also: oskorušica PG	s	r	fr	
*Tanacetum balsamita* L. (Asteraceae) WA71150			2	1	1	4	c	Korčula, Hvar, Zlarin	kalober, kaloper	m	r	l	
*Teucrium montanum* L. (Lamiaceae)	1				1	2	w	Korčula, Krk	trava iva	m	r	ae	
*Teucrium polium* L. (Lamiaceae) WA66373				2		2	w	Brač	trava iva	m	r	ae	
*Thymus longicaulis* C.Presl (Lamiaceae) WA71151	1	1		6	8	16	cw	Brač, Cres, Hvar, Korčula, Pag, Šipan, Vis	majčina dušica, popunac, also: poponac BR	m	r	ae	
*Urtica pilulifera* L. (Urticaceae) WA66441 and Urtica urens L. (Urticaceae) WA66423					1	1	w	Korčula	kopriva	m	r	l	
*Vitex agnus-castus* L. (Lamiaceae) WA66905		1				1	w	Pag	konopljika	m	r	s	
*Vitis* cf. *vinifera* L. (Vitaceae)	3	2	1	2		8	c	Throughout	grožđe (fruit), vinova loza (plant)	s	rb, r, w	fr	
*Ziziphus jujuba* Mill. (Rhamnaceae) WA66442	8	10	1	1	4	24	c	Throughout more in the north	čičinka, čičindra, čičinda, žižula, also: čičimak ŠI, čičindula DU, zizula CR, žužila UG	s	r	fr	

*Two capital letter abbreviations refer to particular islands and are made from the first two letters of their name, e.g. *ZL* for Zlarin and *PA* for Pašman, with an exception for *PG* Pag

1—presence of other species: m – in mixed-species beverages, s – single species flavouring

2—type of use: r – for rakija favouring, rb – as base for rakija fermentation, w – as base for wine-type drinks (fermentation without distillation)

3—part of plant used: ae – aerial parts, fl – flowers, fr – fruit, im – immature fruit, l – leaf, pe – fruit peel, s – seeds, st – stigmas, u – underground parts

The plants most commonly used to flavour drinks are fennel *Foeniculum vulgare* Mill., myrtle *Myrtus communis* L., sage *Salvia officinalis* L., rue *Ruta graveolens* L., juniper *Juniperus oxycedrus* L., carob *Ceratonia siliqua* L., walnut *Juglans regia* L., citrus fruit peel and leaves (mainly lemon *Citrus limon* (L.) Osbeck and bitter orange *Citrus aurantium* L.), fig *Ficus carica* L., bay *Laurus nobilis* L., rosemary *Rosmarinus officinalis* L., wormwood *Artemisia absinthium* L., petals of sweet smelling old rose garden varieties, mainly *Rosa centifolia* L., and mints (mainly *Mentha piperita* L. and *M. spicata* L.). Myrtle, walnut, rose petals and different cherry varieties and species (mainly *Prunus cerasus, P. spinosa* or *P. avium*) are used to make one-species sweetened liqueurs, which contain 20–40% alcohol. Other fruit-based single species sweet spirits are made of jujube *Ziziphus jujuba* L., nettle tree *Celtis australis* L., blackberry *Rubus ulmifolius* Schott, loquat *Eriobotrya japonica* (Thunb.) Lindl., dog rose *Rosa canina* L. and service tree *Sorbus domestica* L.

The remaining species are usually mixed. One kind of mixed-species spirit is called *travarica* (‘trava’ means ‘herb’). The most common ingredients of this drink are fennel, sage, rue, juniper, carob, citrus fruit peel or leaves, bay, rosemary, wormwood, St. John’s wort *Hypericum perforatum* L., savory *Satureja montana* L., wild thyme (*Thymus* spp.), catmint *Clinopodium nepeta* (L.) Kuntze, marjoram *Origanum majorana* L., curry plant *Helichrysum italicum (*Roth) G. Don and lavender (*Lavandula* spp.)*.*

The correlations between the number of species listed by an informant and the studied independent variables were very weak (Table 3). The strongest correlation was found with geographical longitude. Weak negative correlations were found for the age of informants and number of species in the local flora, i.e. younger people and people from more species-poor islands tended to quote more species! Men listed slightly more species than women, but the difference was small and insignificant.

**Table 3:** Correlation matrix for the studied variables. Correlation coefficients are given in the lower left hand corner, the *p* values in the upper right corner

	Number of species	Male gender	Age	Area	Population	Flora	Longitude	Isolation
Number of species		0.06846	0.01497	0.068294	0.33289	0.0023638	7.8724E−06	0.83377
Male gender	0.10678		0.014333	0.08595	0.10751	0.79029	0.10704	0.55088
Age	− 0.14252*	− 0.14367		0.39593	0.13228	0.31776	0.16676	0.87104
Area	− 0.10666	0.10067	− 0.049948		5.5801E−61	6.6006E−40	0.48405	3.7934E−11
Population	− 0.056766	0.094381	− 0.088442	0.77916		4.8659E−32	0.92627	1.4461E−11
Flora	− 0.18295*	0.016168	− 0.060801	0.68899	0.63263		4.3672E−05	0.10277
Longitude	0.25774*	0.094508	− 0.081271	− 0.041041	− 0.005429	− 0.24428		0.00011253
Isolation	0.012313	− 0.035043	0.0095575	− 0.37378	− 0.38116	− 0.098777	− 0.2237	

* significant correlation between a dependent and independent variable, *p* < 0.05

**Fig. 5:**
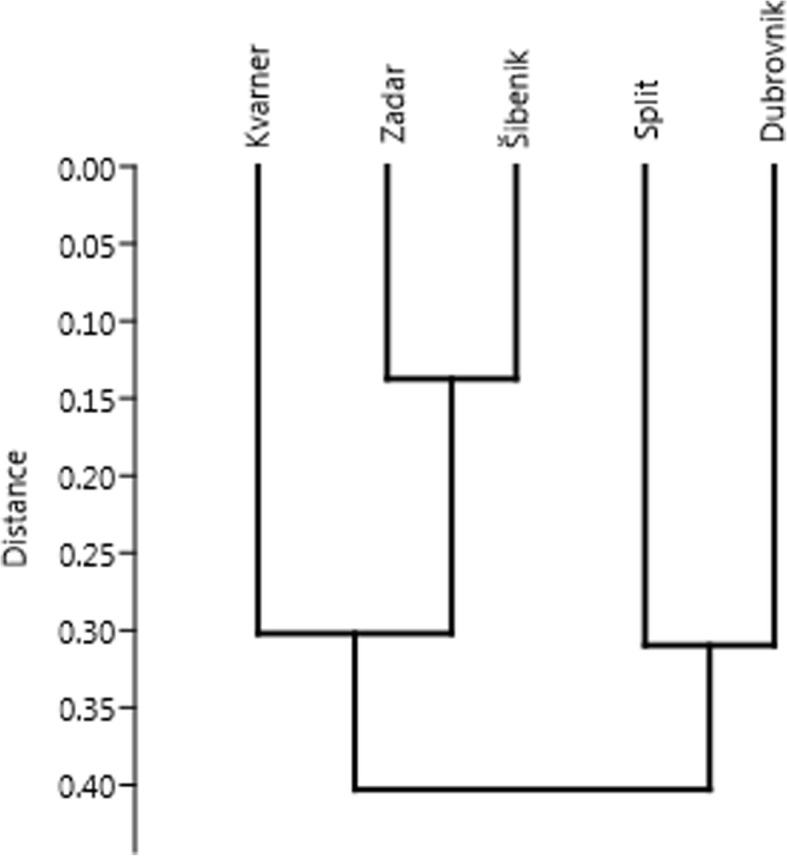
Dendrogram of UPGMA clustering of regions based on the matrix of species used in them to flavour alcoholic drinks

Most of the common ingredients we found are used throughout the Adriatic Islands. On the other hand, some northwest-southeast trends can be detected for a few species, and the number of species per informant was lower in the Kvarner and Zadar archipelago than further south (Fig. 4). The largest number of species per interview was recorded for the Šibenik islands, but it was nearly identical for the Split and Dubrovnik islands as well (Fig. 4). In terms of species composition, clustering differentiated the archipelagos into two groups, one constituted by the Dubrovnik and Split islands and the other by the Kvarner, Zadar and Šibenik islands. Thus, the Šibenik islands have an intermediate character—people use as many species as they do further south, but the species composition is closer to the more northern Kvarner and Zadar islands (Fig. 5).

**Fig. 6:**
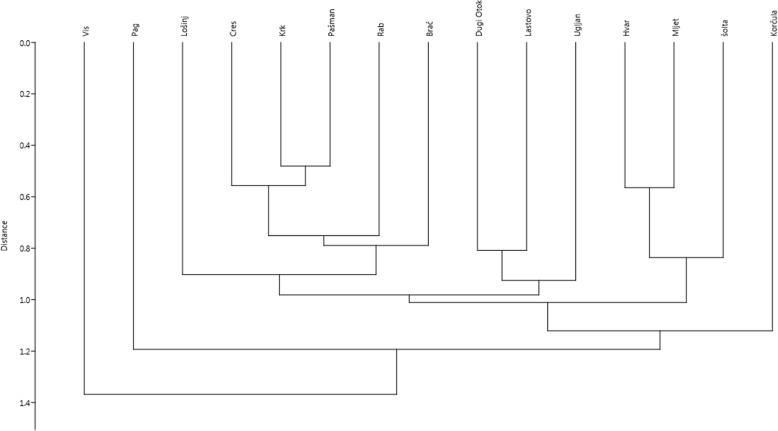
Dendrogram of UPGMA clustering of the largest 15 islands based on the matrix of species used on them to flavour alcoholic drinks

When each island was analysed separately the largest number of taxa was recorded in Korčula (49), Vis (39), Zlarin (37), Hvar (30) and Šipan (27). The clustering analysis revealed that Vis, Pag and Korčula were the least similar to other large islands (Fig. 6). During our interviews, we noticed a high level of experimentation. People often mentioned trying new plants in search of interesting flavours. As many as 42 taxa were mentioned by only one or two informants, which show a high level of idiosyncrasy in the species composition used for rakija. However, there are a few species which are both restricted to one island and constitute a salient part of local culture. One example is rakija flavoured with *Artemisia caerulescens* L. made on Cres. The plant is nearly extinct now and the drink is made only by a few people on the island, as the species is difficult to find (this case is discussed in more detail later in this chapter). Another example is the use of grey rock-rose *Cistus* × *incanus* L. and *Rhamnus alaternus* L. in travarica mixes, which is restricted only to the island of Vis. These plants are very common on most of the islands [46], but are not used there. A similar situation occurs on Brač where the uses of *Micromeria juliana* (L.) Benth. ex Rchb. and felty germander *Teucrium polium* L. were recorded, but the species are found on other islands as well.

*Citrus × aurantium* L., bitter orange, is characteristic for the former Republic of Dubrovnik (now the Dubrovnik region including the studied islands from this area), where it was introduced in the tenth century. It has a special place in gardens, summer houses and monasteries in the area of Dubrovnik. Bitter orange is often used in local gastronomy, as a spice and for medicinal purposes, and it is also an indispensable ingredient of the Dubrovnik ‘travarica’. Interestingly, further north in other parts of Dalmatia (e.g. near Split), sweet orange *Citrus sinensis* (L.) Osbeck is used more often than bitter orange. This difference has only a cultural or historical and not climatic explanation.

Among interesting plants occasionally used in rakija, we must mention lemon verbena, *Aloysia citriodora* Palau, which is used mainly on Korčula. According to local beliefs recorded in this study, the plant was brought by sailors to the island, directly from South America.

The recreational and digestive uses of plant flavoured alcohols are dominant, and they are rarely used for particular ailments. Digestive stomachic properties are generally attributed to mixed-species travarica. Out of all the herbal grappas, rue travarica is thought to be the most powerful. It is particularly used to increase appetite and prevent the *evil eye* (‘evil eye’ is a folk illness—it was commonly believed throughout Europe that a certain kind of person may hurt others, animals and crops, just by looking at them). The consumption or wearing of certain plants had a protective function against those with the ‘evil eye’ [8, 51]. Other common stomachics are walnut (unripe fruit) and wormwood grappas. An interesting tradition persists in the Kvarner islands, where a sweet medicinal liqueur made from bay fruits is widely known.

Many of the species used to flavour alcoholic drinks are the same as those used on the other side of the Adriatic, in central Italy (sour cherries, walnuts, citrus fruits and many wild herbs such as sage) [1, 2, 52]. Thirty-two, i.e. nearly half of the wild plants recorded in the study, are also used for making liqueurs in Spain (including Catalonia) [7]. In Spain, similarly to the results of our study, Lamiaceae and Rosaceae are the families containing the largest number of plants used as liqueur ingredients. The similarity would be larger if it were not for the fact that in some cases different species from the same genus are used in the different countries (i.e. *Thymus*, *Ruta*, *Pyrus*). It is striking that in Croatia use of foreign (i.e. imported) ingredients in rakija is nearly non-existent. Practically all the plants used for flavouring come from home gardens or the surrounding nature and are usually self-collected. Not a single species of imported spices is used (apart from coffee), in contrast to many other areas of Europe where spices such as star anise *Illicium* spp. or cloves *Syzygium aromaticum* (L.) Merrill & Perry and cinnamon *Cinnamomum* spp. have been common ingredients of alcoholic drinks for a long time [15]. Strangely, the Venetians, who ruled the Croatian islands for hundreds of years, were some of the main traders of exotic spices [15]. In spite of this, the custom of combining them with alcohol did not permeate to the island culture. Locally grown Mediterranean spices, such as marjoram, anise and saffron, are also used sparingly. This avoidance of exotic imported spices in travarica goes along with the general trend of avoiding spices in south Croatian cuisine, particularly in Dalmatia [53]. Croatian cooking uses spices mainly for fish and game dishes (rosemary) or for preserving fruits and marinades (rosemary, bay leaf). Both rosemary and bay leaf are of local origin. Even native oregano and thyme species have not been, according to our respondents, used in cooking until recently. Making high alcohol spirits distilled from fruits must have a history stretching back a few hundred years in Croatia, and our respondents remember flavouring rakija with single species of mixed herbs since their childhood. However, they observed that making multi-species aromatic travarica has intensified as a result of the development of tourism since the 1960s. Before this time, single species flavouring was more widespread (rue, walnut, sour cherry, sage, myrtle). Another change has occurred in the way travarica is made. When people commonly distilled their rakija at home, herbs were added to the fermented fruits. Nowadays, they are more commonly added to bottles after the distillation process. In contrast to the thriving tradition of travarica, the use of local low-alcohol drinks from the pseudofruits of juniper (recorded on Rab and Cres, as well as on the mainland of Croatia in Istria [24]) has completely disappeared.

Some new fashions may be observed, particularly making rakija with ripe olive fruits in it. The practice of adding exotic fruit species to rakija, mainly jujube and loquat, has also increased in frequency, though it was already present throughout the islands a few decades ago.

The incredible richness of species used in the Adriatic islands should be emphasised and treated as a local cultural heritage. In a very comprehensive review of plants used to make alcoholic drinks in the whole of Eastern Europe, 116 species were listed [6], whereas we recorded nearly as many as 114 in one part of one small country! In contrast, in a region of Italy as many as 46 species were recorded [2], which is still treated as a rich heritage. Comparable species richness was only observed in beer starters in a Shui minority area in Guizhou, China, where 102 species are used [12]. A large number of species used to produce home-made liqueurs is also used in Spain (including Catalonia), where at least 125 species of exclusively wild plants have been recorded [7], not taking into account cultivated species.

Although a rich tradition of flavouring strong spirits distilled from grape pomace is alive and even expanding, some culinary phenomena, such as the distillation of *Arbutus unedo* fruits, and making wine with *Juniperus* pseudo-fruits, are now extinct. These two beverages might be revived by local family farmers on a wave of general public interest in minor non-timber forest products [54]. It is worth emphasising that *Juniperus* spp. are spreading on land which once used to be grazed; thus, the utilisation of their fruits could increase local ecosystem services. The use of juniper beverages has been declining, in the Croatian islands as well as in northern Europe, with an exception of very few locations, e.g. Kurpie, Poland, where it has revived [9]. Madej et al. [9] claim that fermented juniper drinks have a very long history in Europe, of at least a few thousand years, based on archaeological findings from Denmark where ancient sediments of such a drink were found [55].

The common use of a large number of ingredients in rakija is probably a symptom of the gentrification of local gastronomic traditions. The term gentrification was first applied by the sociologist Ruth Glass in 1964 to ordinary, working-class areas of the city of London that became increasingly inhabited by middle-class people, artists etc. [56]. Now, the term is sometimes extended to cuisine as well. In contemporary times, we eagerly search for new ingredients and experiment with them [57]. Wild plants nowadays often become delicatessen as noted by the authors from the Iberian Peninsula [58]. They have also become an important part of menus in expensive restaurants [59]. In a tourism-oriented area of the Adriatic islands home-made spirits are often sold to visitors; thus, the pressure to impress tourists and provide an interesting product for them must have been an additional driving force towards experimentation with flavourings in alcohol. Interestingly, however, the ingredients used are nearly exclusively local, whether wild or grown. This is in contrast to other European countries, in which people often experiment with exotic spices. The Croatian islands formed part of the Venetian empire for a long period of time. The Venetians were some of the leading distilled alcohol producers in Europe. We are convinced that most of the flavouring ingredients we noted have a long history of local use in alcoholic drinks, but it used to be restricted to medicinal uses or used only by noblemen, and has only slowly become widespread and common. These species are usually used to make herbal teas as well; thus, it is likely that local people automatically switched from using the same species in herbal teas to using them in alcohol. The same likely trajectory was discussed for the popularisation of *genepì* in the western Alps. The name *genepì* is applied in the Western Alps (in Occitan, Franco-Provencal, and in French and Italian too) to diverse species of locally growing Alpine wormwood (esp. *Artemisia genipi, A. glacialis, A. umbelliformis*), which were once used locally as medicinal herbs, and later became part of a sought-after alcoholic drink [60]. These species of *Artemisia* have been under threat of over-collection since the nineteenth century [61]. In our study, most of the plants used as flavouring are common or cultivated, apart from one which may be threatened. This is also an *Artemisia* species, bluish-leaved wormwood *A. caerulescens*. The species has a few dozen localities in coastal Croatia, restricted mainly to salt marshes, and is not very abundant [46]. The history of the interest in the utilisation of the species in the northern Adriatic is a long one [62]. It is only collected on the island of Cres, where it is known as *morski pelin* (‘sea wormwood’), although the Venetian-origin name *sandonego* is also known. The local inhabitants are aware of the Venetian roots of the drink they make from it. According to our informants in Cres, it has been severely overharvested and practically destroyed by gathering and the development of tourist facilities on the shores, and it is now on the verge of extinction.

*A. caerulescens* and *Satureja visianii* Šilić seem to be the only relatively rare aromatic plant used. Other species from the impressive list of ingredients are common in the wild or commonly cultivated on the Croatian islands. Perhaps, this is what enabled a negative correlation between the number of species listed and the flora of the island (Table 3).

Starting the research, we expected a set number of species to be used in the multispecies rakijas. Certain numbers, such as 3, 7 and their multiplications (9, 12, 27) have positive connotations in European culture [8, 63], and one of the authors observed that some people in northern Croatia use a set of 12 species for making rakija. However, here on the islands, no such customs were observed.

## Conclusions

The study showed that the Croatian islands host a rich tradition of flavouring spirits. In terms of species-richness, this is, to the best of our knowledge, one of the longest lists of species used for flavouring alcoholic drinks ever recorded. Another interesting finding is the relic tradition of making a low-alcohol juniper beverage, mainly on the islands of the Kvarner archipelago.

## Data Availability

The data matrix analysed during the current study is available upon request. Voucher specimens for species were deposited in the herbariums of Warsaw University (WAW) and the University of Zagreb (ZAGR).
